# Pathoadaptive evolution and clonal dissemination of community- associated methicillin-resistant *Staphylococcus aureus* in Egypt

**DOI:** 10.1186/s12879-026-13097-w

**Published:** 2026-04-02

**Authors:** Nehal A. Saif, Reem A. Elghaish, Eman Badr, Shaimaa F. Mouftah, Sherine M. Shawky, Ben Pascoe, Samuel K. Sheppard, Mohamed Elhadidy

**Affiliations:** 1 Center for Genomics, Helmy Institute for Medical Sciences, Zewail City of Science and Technology, Giza, Egypt; 2 Biomedical Sciences Program, University of Science and Technology, Zewail City of Science and Technology, Giza, Egypt; 3 Faculty of Computers and Artificial Intelligence, Cairo University, Giza, Egypt; 4 Department of Microbiology, Medical Research Institute, Alexandria University, Alexandria, Egypt; 5 Ineos Oxford Institute for Antimicrobial Research, Department of Biology, University of Oxford, Oxford, UK; 6 Department of Bacteriology, Mycology, and Immunology, Faculty of Veterinary Medicine, Mansoura University, Mansoura, Egypt

**Keywords:** Community-acquired MRSA (CA-MRSA), Antimicrobial resistance, Clonal dissemination, Resistome, Virulome, Genomic adaptation

## Abstract

**Background:**

*Staphylococcus aureus* is a major public health concern and is classified as a priority pathogen by the World Health Organization (WHO) with the global rise of methicillin-resistant *S. aureus* (MRSA) infections. Community-associated MRSA (CA-MRSA) strains have become increasingly important in both community and healthcare settings. This study aimed to investigate the genomic diversity, evolution, resistome, and virulome of CA-MRSA isolates circulating in Egypt to better understand their persistence, adaptation, and public health implications.

**Methods:**

A total of 123 CA-MRSA isolates were collected from clinical settings in Alexandria, Egypt. Methicillin resistance was first determined phenotypically using cefoxitin resistance, followed by genotypic confirmation through detection of the *mecA* gene.Whole-genome sequencing and comparative genomic analyses were performed to characterize sequence types, clonal complexes, SCC*mec* elements, resistance determinants, and virulence factors. Phylogenetic relationships were reconstructed to assess evolutionary divergence, and network analysis was used to explore associations between resistance and virulence gene profiles.

**Results:**

Eight distinct clonal complexes (CCs) were identified, dominated by CC121-SCC*mec*V (15%), CC1-SCC*mec*V (14%), CC15-SCC*mec*V (9%), CC1-SCC*mec*VI (7%), and CC8-SCC*mec*V (6%). Five novel sequence types (ST8157–ST8161) were discovered and deposited in pubMLST, indicating ongoing local evolution. Within CC8, two divergent lineages (ST239 and ST8) harbored unique SCC*mec* elements, reflecting significant phylogenetic differentiation. Globally important epidemic clones such as ST239-III-MRSA and ST22-IV-MRSA (EMRSA-15) were also detected. Network analysis revealed broad ecological adaptability, with livestock-associated CC97 and healthcare-associated CC5 harboring genes for immune evasion and biofilm formation. The detection of *yopB* in CC97 and *yscT* in CC5, genes typically found in *Yersinia* species, suggests horizontal gene transfer as a mechanism of adaptation. The high prevalence of *fosB* (fosfomycin resistance) and elevated fusidic acid resistance (39%) further underscores the emergence of multidrug resistance.

**Conclusions:**

This large-scale genomic analysis reveals the coexistence of globally disseminated and locally evolved CA-MRSA lineages in Egypt. The findings underscore the adaptive potential of Egyptian MRSA populations and their contribution to regional AMR dynamics. Continued genomic surveillance within a One Health framework is essential for monitoring MRSA evolution, informing control measures, and mitigating the spread of resistance in both community and clinical settings.

**Clinical trial:**

Not applicable.

**Supplementary Information:**

The online version contains supplementary material available at 10.1186/s12879-026-13097-w.

## Introduction

Antimicrobial resistance (AMR) is one of the most pressing global health challenges of the 21st century, threatening the effective treatment of infectious diseases worldwide [1, 2]. In Egypt and the broader Eastern Mediterranean region, AMR is alarmingly high, driven by weak healthcare infrastructure, unregulated antibiotic use, and limited national stewardship and surveillance capacity [3]. The problem extends beyond hospitals, with resistance circulating among community and livestock-associated bacteria, underscoring the need for an integrated One-Health approach to address high-priority pathogens [4], including the ESKAPE group (*Enterococcus faecium*, *Staphylococcus aureus*, *Klebsiella pneumoniae*, *Acinetobacter baumannii*, *Pseudomonas aeruginosa*, and *Enterobacter* species), which are major contributors to drug-resistant infections worldwide [5].

*Staphylococcus aureus (S. aureus)* is a major opportunistic pathogen colonizing humans and animals [6, 7]. It causes a broad spectrum of infections, ranging from skin and soft tissue infections to life-threatening bacteremia, and food-borne intoxication [6, 8]. Methicillin-resistant *S. aureus* (MRSA) strains, characterized by the *mecA* gene carried on the mobile staphylococcal cassette chromosome (*SCCmec*), are of particular concern and have been designated by the World Health Organization (WHO) as priority pathogens [1, 9].

The adaptability of MRSA has enabled its global spread [10, 11]. Initially confined to healthcare settings (HA-MRSA), MRSA has become established in community-associated infections (CA-MRSA) [12, 13]. CA-MRSA strains, often carrying *SCCmec* types IV or V and virulence factors such as Panton-Valentine leukocidin (PVL), are typically more transmissible and can replace traditional HA-MRSA lineages in both hospitals and the community. *S. aureus* harbors the accessory gene regulator (Agr) operon, a quorum-sensing (QS) system that controls the expression of numerous virulence factors. Genetic variability within the Agr locus generates diverse phenotypic traits among bacterial populations, promoting adaptation and survival across different environments. A functional Agr system appears to play a critical role in the virulence of CA-MRSA, in contrast to HA-MRSA [14, 15]. The rise of livestock-associated MRSA (LA-MRSA), particularly clonal complex CC97, has further blurred these epidemiological boundaries, reflecting frequent host adaptation and zoonotic transmission [16–18].

The global population structure of *S. aureus* includes several dominant clones that vary in prevalence by region and time [19, 20]. While HA-MRSA lineages such as ST239 and ST22 predominate in Asia and Europe, CA-MRSA lineages including ST93, ST772, and ST80 are widespread in Australia, South Asia, and North Africa [1, 21, 22]. In Egypt, limited genomic studies have reported the co-circulation of both HA- and CA-MRSA clones (e.g. CC5, CC22, CC30, CC80, CC1), but the evolutionary context and diversity of community strains remain poorly defined [23, 24].

This study provides a cross-sectional genomic snapshot of 123 CA-MRSA isolates collected from Alexandria, Egypt. While not a formal surveillance program, these data offer a valuable baseline for understanding the diversity, population structure, resistome, virulome, and evolutionary dynamics of CA-MRSA circulating in North Africa. Using comparative genomics and phylogenetic analysis, we investigate how these lineages relate to global MRSA clones and identify genetic factors contributing to their adaptation and persistence across ecological niches.

## Methods

### Isolate collection

**Fig. 1:**
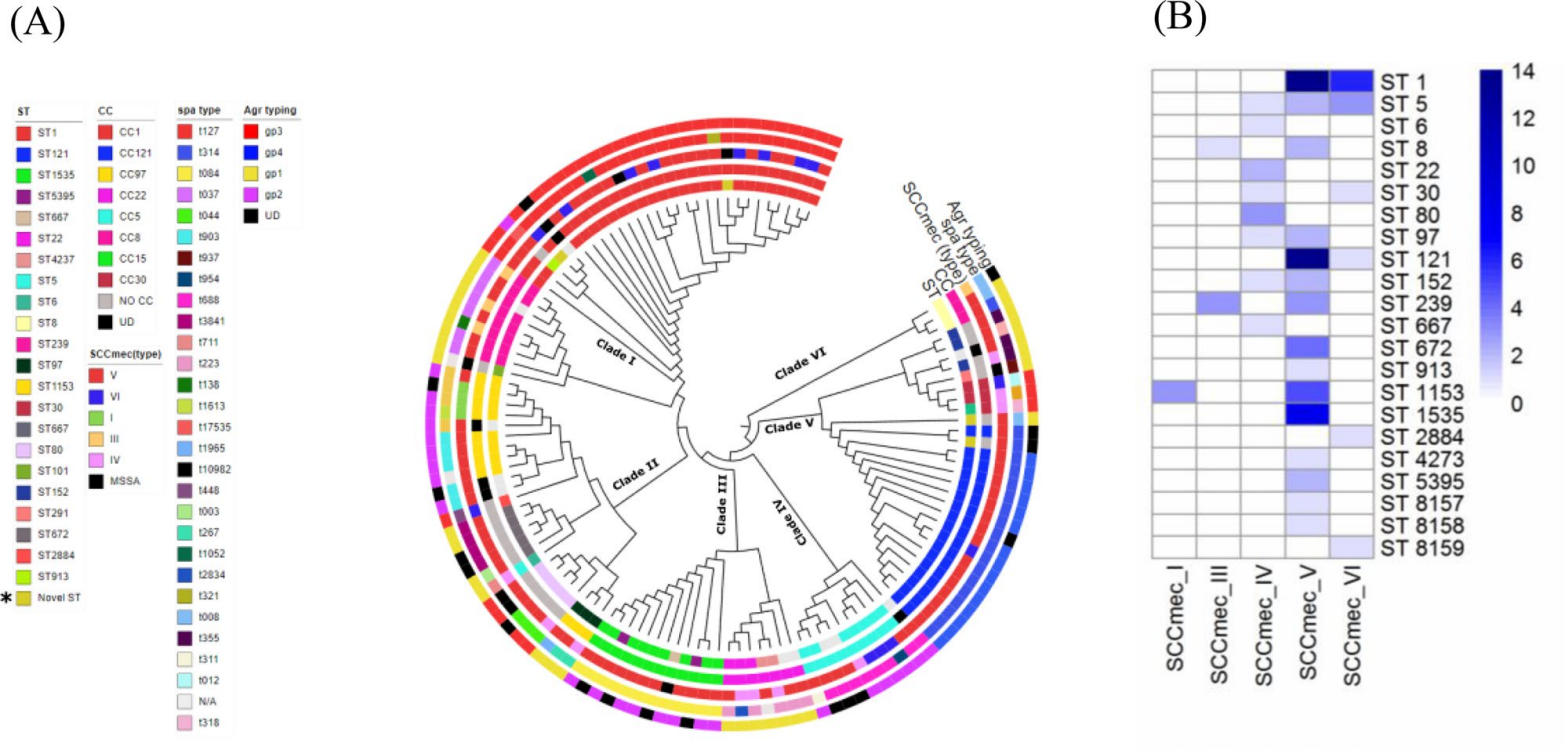
(**A**): Core genome maximum-likelihood (ML) cladogram of 123 MRSA isolates based on an alignment of 1,347 core genes.The cladogram illustrates tree topology, and concentric rings (inner to outer) indicate sequence type (ST), clonal complex (CC), SCC*mec* type, *spa* type, and *agr* type. UD = undetermined; N/A = not available. Six clades were identified: Clade I (CC1/SCC*mec*V-VI and CC8/SCC*mec*III-V), Clade II (CC97-SCC*mec* I-IV-V), Clade III (CC15-SCC*mec*V and CC22-SCC*mec*IV-V), Clade IV (CC5-SCC*mec*IV-V-VI), Clade V (CC121SCC*mec*V-VI and CC30-SCC*mec*VI-IV), and Clade VI (CC8- SCC*mec*III-V). (**B**): Distribution of SCC*mec*types among CA-MRSA sequence types (STs) in Egypt. The numbers on the right indicate the total counts of SCC*mec* types observed within each ST. Darker shades represent higher frequencies, emphasizing clonal diversity and the prevalence of specific SCC*mec* elements among CA-MRSA strains in Egypt

A total of 123 representative non-duplicate *S. aureus* isolates were collected from a clinical diagnostic center serving multiple screening laboratories in Alexandria, Egypt, between August 2020 and April 2021 (Fig. 1A). The isolates were collected before the study period as part of historical sampling; therefore, ethics approval and patient consent were not required. To ensure diversity and prevent the inclusion of closely related isolates, we employed random sampling stratified by month of isolation and sample source. Isolates were recovered from different clinical specimens, including aspirate (*n* = 44), wound (*n* = 20), pus (*n* = 26), abscess (*n* = 8), mini-bronchoalveolar lavage (miniBal) (*n* = 4), blood (*n* = 3), sputum (*n* = 3), catheters (*n* = 3), and peritoneal fluid (*n* = 1).

Species confirmation was performed using previously described phenotypic, morphological, and biochemical markers [25]. All isolates were further confirmed as * S. aureus* by molecular detection of the thermonuclease (*nuc*) gene (Supplementary File 11).

Methicillin resistance was initially determined phenotypically based on resistance to cefoxitin according to CLSI guidelines (CLSI M100). Genotypic characterization of methicillin resistance was subsequently performed by screening for the *mecA* gene [26]. Isolates were classified as community-acquired MRSA (CA-MRSA) based on the standard epidemiological criteria of the US Centers for Disease Control and Prevention (CDC), including infection onset in outpatient settings or within 48 h of hospital admission in patients with no prior healthcare exposure, in combination with phenotypic evidence of methicillin resistance [27]. Molecular detection of *mecA* was used to further characterize methicillin resistance mechanisms but was not the sole criterion for CA-MRSA designation. All isolates were stored at − 80 °C in brain heart infusion broth supplemented with 15% (v/v) glycerol for further analysis.

### Antimicrobial susceptibility testing

The antimicrobial susceptibilities of all MRSA isolates were detected using the Kirby-Bauer disk diffusion method on Mueller–Hinton agar plates (HiMedia Laboratories Pvt Ltd., Mumbai), following the guidelines of the Clinical and Laboratory Standards Institute (CLSI) [28]. The antibiotics used in this study are categorized by class as follows: Aminoglycosides, including amikacin (30 mcg), tobramycin (10 mcg), and gentamicin (10 mcg); Beta-Lactam/Beta-Lactamase Inhibitors, including amoxicillin-clavulanic acid (30 mcg), ampicillin (10 mcg), ampicillin-sulbactam (10 mcg), and tazobactam-piperacillin (30 mcg); Cephalosporins, including cefadroxil (30 mcg) as a first-generation agent, cefuroxime sodium (30 mcg) as a second-generation agent, cefotaxime (30 mcg), ceftazidime (10 mcg), ceftriaxone (30 mcg), and cefoperazone (75 mcg) as third-generation agents, cefepime (30 mcg) as a fourth-generation agent, and cefoxitin (30 mcg) as a cephamycin, with cefoperazone-sulbactam (30 mcg) included as a third-generation cephalosporin with a beta-lactamase inhibitor; Folate Pathway Inhibitors, including trimethoprim-sulfamethoxazole (25 mcg); Fluoroquinolones, including ciprofloxacin (5 mcg), moxifloxacin (5 mcg), ofloxacin (5 mcg), and levofloxacin (5 mcg); Macrolides, including erythromycin (15 mcg), clarithromycin (15 mcg), and azithromycin (15 mcg); Lincosamides, including clindamycin (2 mcg) and lincomycin (15 mcg); Tetracyclines, including doxycycline (30 mcg), minocycline (30 mcg), and tigecycline (15 mcg) as a glycylcycline derivative; Carbapenems, including imipenem (10 mcg), meropenem (10 mcg), and ertapenem (10 mcg); Fusidanes, including fusidic acid (10 mcg); Oxazolidinones, including linezolid (30 mcg); Rifamycins, including rifampicin (15 mcg); and Glycopeptides, including teicoplanin (30 mcg) and vancomycin (30 mcg).

The screened isolates were classified as susceptible or resistant based on their zones of inhibition and were defined as MDR if they exhibited resistance to at least one agent in three or more antimicrobial classes [29]. *S. aureus* ATCC 25923 was used as a quality control strain.

### DNA extraction and whole genome sequencing

The QIAamp DNA Mini Kit (Qiagen, UK) was used to extract genomic DNA from MRSA isolates according to the manufacturer’s instructions, with some modifications. These included increasing the initial volume of bacterial suspension to 2 mL and warming the elution buffer to 60 ℃. Additionally, 200 µL of 20 mg/ml lysostaphin (Sigma-Aldrich, USA) containing lysis buffer was added to facilitate cell lysis. DNA was eluted in 100 µl of elution buffer and quantified using a Nanodrop spectrophotometer (Thermo Fisher Scientific, USA).

Libraries were prepared using Nextera XT kits (Illumina, San Diego, CA) according to the manufacturer’s instructions, with 48 isolated gDNA samples per batch, each barcoded. MiSeq run kits (v3) were used to generate 2 × 300 base paired-ends on Illumina’s MiSeq sequencing platform. Quality control, adapter trimming, and quality filtering of the FASTQ data were performed using Fastp (v0.23.2) command line tool, with the activated error correction option using the C parameter to perform overlap analysis for paired-end reads [30]. *De novo* genome assembly was conducted using SPAdes (version 3.13.0) with the careful command [31]. Assembly statistics were calculated on the filtered assembly with QUAST 4.4 [32], and the assemblies were polished 5 times using Pilon (v1.24) [33]. The average size of the polished assemblies was 2.71 ± 0.4 Mbp (Supplementary file 1). The assembled contigs were processed using Prokka (v1.14.5) [34]. For the pangenome analysis of the MRSA isolates, PEPPAN (v1.0.5) [35], an integrated computational tool that accurately delineates core and accessory gene content, was employed.

### Annotation and typing

Prokka was run with default parameters to ensure consistency across all genomes, generating standardized GenBank-formatted output files for downstream analysis [34]. FastMLST (v0.0.15), a multilocus sequence typing (MLST) tool that uses BLASTn to perform PubMLST searches [36], was used to determine the sequence type (ST), allelic profile and clonal complex (CC) for the draft assemblies [36]. Clonal complexes (CCs) were defined based on sharing five or more alleles with a central genotype (ST) [37]. The Staphopia-sccm (v1.0) command-line tool was executed to determine the SCC*mec* types of the screened isolates from their draft assemblies [38]. Additionally, SpaTyper (https://github.com/HCGB-IGTP/spaTyper, Version 0.3.3) was used to generate spa type identification from the draft assemblies. AgrVATE (Version v1.0.0, https://github.com/VishnuRaghuram94/AgrVATE) was employed to identify *agr* locus types and variants from draft assemblies, using its k-mer-based approach to assign *agr* groups efficiently and consistently.

### Core and accessory genome characterization

Core and accessory genomes were determined using PEPPAN(v1.0.5), a robust pangenomics pipeline that combines tree- and synteny-based approaches to provide consistent genome annotations while excluding paralogous genes [35]. Core genes were defined as those present in at least 95% of the genomes analyzed, ensuring a stringent threshold for conserved genes across the dataset [35]. ABRicate (v0.7) was used to screen the assemblies for AMR genes with NCBI Bacterial Antimicrobial Resistance Reference Gene Database, along with ResFinder database [39, 40]. Additionally, ABRicate was used to screen for virulence factors (VFs) using the Virulence Factor Database (VFDB) [39, 41].

The phenotypic resistance of MRSA isolates was compared with genomic predictions of AMR based on their assembled genomes. The concordance rate was calculated as the percentage of isolates showing agreement between phenotypic resistance and the presence of corresponding resistance determinants in their genomes, relative to the total number of isolates [42]. To better characterize genotype-phenotype mismatches, we categorized discordance into two distinct types: (Type 1) isolates exhibiting phenotypic resistance without a corresponding resistance gene, and (Type 2) isolates carrying a resistance gene without associated phenotypic resistance.

### Comparison of core and accessory genome phylogenies

Parsnp from Harvest v1.2 was utilized to construct core genome alignments [43].The contiguous non-conserved regions of the alignment file were removed using Gblocks v0.91b [44]. To construct the maximum-likelihood (ML) phylogenetic tree, the RAxML-HPC v8 command-line tool (under the GTR+GAMMA model) was employed, using a general time-reversible model, a GAMMA substitution rate, and rapid bootstrapping (*n* = 1000). The resultant RaxML best tree and its metadata were visualized using the iTOL tool with branch lengths reflecting evolutionary distances (substitutions per site) [45].

To analyze evolutionary relationships, we first constructed a phylogenetic tree from the gene presence matrix using the pangenome and the hclust function in R. This tree was then compared with a core genome phylogenetic tree generated with RAxML. To visualize and compare the two trees, we employed the cophylo function from the phytools package (Version 1.9), which enabled us to construct and interpret the co-phylogeny between the gene-absence matrix and the core-genome data.

To evaluate the association between CCs and the presence of specific AMR genes, as well as between CCs and phenotypic resistance profiles, Fisher’s exact test was performed using RStudio (version 1.3.1093). For each resistance gene, a 2×n contingency table was constructed to compare gene presence/absence across the different CCs. The fisher.test() function in R was used to compute p-values and odds ratios (ORs), enabling the identification of statistically significant associations between specific CCs and resistance determinants. Results were summarized for each gene, highlighting those whose distribution was significantly associated with CCs.

To assess overall differences in the number of AMR and virulence factor (VF) genes across CCs, we used the Kruskal–Wallis test, a non-parametric method appropriate for comparing distributions across multiple independent groups. For post hoc analysis, pairwise Wilcoxon rank-sum tests were conducted with Holm correction to control the family-wise error rate. p-values were adjusted for multiple comparisons using the Benjamini–Hochberg false discovery rate (FDR) procedure. Adjusted p-values were used to detect statistically significant differences in AMR gene counts and to annotate the plots. To integrate phylogenetic and statistical insights, a phylogenetic tree of the analyzed isolates was constructed and overlaid with horizontal boxplots illustrating the distribution of VF and AMR gene counts across CCs. Statistically significant pairwise comparisons from the Wilcoxon tests were annotated directly on the plots using brackets and asterisks to indicate the degree of significance. To reduce the influence of within-clone redundancy, comparisons of AMR and virulence gene counts were performed at the CC level, providing a lineage-based rather than isolate-based assessment of gene distribution. This integrative approach provided a comprehensive view of the phylogenetic relationships alongside clonal lineage-specific trends in virulence and resistance gene content.

To investigate the distribution of virulence genes across clonal complexes (CCs), we generated a presence/absence matrix of virulence genes across all isolates and constructed a bipartite network linking virulence genes to CCs based on shared presence. To enhance visualization clarity, we retained only genes present in at least two of the four selected CCs (CC1, CC5, CC121, and CC97). The network was visualized using Cytoscape (version 3.10.3), where nodes represent either virulence genes or CCs, and edges indicate the presence of a given gene in multiple CCs [46].The resulting visualization highlights co-occurrence patterns of virulence genes across lineages, without inferring directionality or evolutionary mechanisms (e.g., horizontal gene transfer or gene loss). The four CCs shown in Fig. 5B were selected based on their relative abundance and diversity of virulence gene content in our dataset. This approach was intended to illustrate general trends and variability, rather than exhaustively represent all lineages.

## Results

### Globally disseminated CA-MRSA clones are present in Alexandria, Egypt

Between August 2020 and April 2021, a total of 123 CA-MRSA isolates were collected from various clinical samples in Alexandria, Egypt (Fig. 1.A). The average genome size of the MRSA isolates was 2.71 ± 0.4 Mbp, with a GC content of 33.31% (Supplementary file 1). The N50 values of the genome assemblies ranged from 1696 bp to 1,028,515 bp, and the number of contigs varied between 29 and 2986. Six clades were identified in the phylogenetic tree: Clade I (CC1-SCC*mec*V-VI and CC8-SCC*mec*III-V), Clade II (CC97-SCC*mec* I-IV-V), Clade III (CC15-SCC*mec*V and CC22-SCC*mec*IV-V), Clade IV (CC5-SCC*mec*IV-V-VI), Clade V (CC121-SCC*mec*V-VI and CC30-SCC*mec*VI-IV), and Clade VI (CC8- SCC*mec*III-V) (Fig. 1.A). The cladogram topology was supported by 1,000 bootstrap replicates. A total of 23 distinct sequence types (STs) were identified (Fig. 1.A), with the most prevalent being ST1-V (*n* = 14; 11%), ST5-V (*n* = 6; 5%), and ST121-V (*n* = 15; 12%). The remaining isolates were distributed across various other sporadic STs (Supplementary file 3).

### Genetic diversity and novel lineages of CA-MRSA

Our analysis identified five novel STs with unique allele profiles, as designated by PubMLST [47]. These new STs included ST8157-V-t314, ST8158-V-t008, ST8159-VI-t127, ST8160-V-t127, and ST8161-V-t084. Notably, these STs represent the first recorded instances in Africa and are predominantly associated with SCC*mec* type V (CC121-SCC*mec*V), except for ST8159, which carries SCC*mec* type VI (ST8159-CC1) (Fig. 1B, Supplementary File 8). Allelic profile comparisons showed that several of the novel STs were single or double-locus variants of known STs, particularly within CC1 and CC121. Core genome and MLST-based clustering further confirmed their close relationship to ST1 and ST121, suggesting these novel STs are regional variants of established CA-MRSA lineages. Among the screened isolates, 25 could not be assigned definitive sequence types because one or two MLST housekeeping gene alleles were partially recovered or absent from the draft genome assemblies, likely due to assembly fragmentation or coverage limitations. The isolates were grouped into eight distinct CCs, including CC1 (ST1; *n* = 22) and CC121 (ST121; *n* = 17). Other major complexes included CC15 (ST1535 and ST5395; *n* = 11), CC8 (ST8 and ST239; *n* = 9), and CC97 (ST97 and ST1153; *n* = 11). Other notable CCs included CC5 (ST5, *n* = 7), CC22 (ST22 and ST4273, *n* = 4), and CC30 (ST30 and ST667, *n* = 3). Thirteen isolates could not be assigned to any specific clonal complex.

A total of 32 distinct *spa* types were detected, with t127 the most prevalent (*n* = 26; 21%), followed by t314 (*n* = 19; 15%), t084 (*n* = 11; 9%), and t668 (*n* = 8; 7%). SCC*mec* typing revealed that most isolates carried SCC*mec*V (*n* = 76; 70%), with other common types including SCC*mec*IV (*n* = 3; 8%), SCC*mec*I (*n* = 3; 2%), SCC*mec*II (*n* = 8; 7%), and SCC*mec*III (*n* = 4; 3%). SCC*mec* type VI was present in 11% of the isolates (*n* = 14). Sequence types attributed to community clones, such as ST1 and ST121, contained the highest number of SCC*mec*IV/V elements (16% and 12%, respectively). In contrast, ST5, typically associated with HA-MRSA [48] contained a combination of SCC*mec*IV/V/VI, a pattern uncommon in typical HA-MRSA. ST97 contained both SCC*mec*II and SCC*mec*V elements. The SCC*mec*V element was identified in local Egyptian STs, ST8157 and ST8158, while ST8159 was associated with SCC*mec*VI, a recently discovered element [49]. Overall, SCC*mec*VI was identified in six distinct STs among the isolate collection.

Our study highlighted a high prevalence of globally disseminated CA-MRSA lineages, particularly CC121-SCC*mec*V (18%), CC1-SCC*mec*V (17%), and CC15-SCC*mec*V (9%). Livestock-associated clonal complexes were also relatively common (CC97-SCC*mec*V at 6% and CC22-SCC*mec*V) as well as hospital-associated lineages in the community isolates (CC5-SCC*mec*V, CC8-ST239-SCC*mec*III). Among the CC8 isolates, two distinct lineages were identified based on core genome SNPs: ST239 (54.5%) and ST8 (27.27%). The *agr* gene typing revealed distinct profiles across clonal complexes. The *agr* group I (*agr* I) was populated by isolates from clonal complexes CC22, CC8, CC97, CC121, and CC5, which are primarily HA clones. The *agr* group II (*agr* II) included isolates from the LA-MRSA lineages CC97, CC5, and CC15. Groups III and IV were linked to CA-MRSA isolates, with CC1 and CC30 in Group III, and CC121 in Group IV.

### Widespread antibiotic resistance among CA-MRSA isolates

**Fig. 2:**
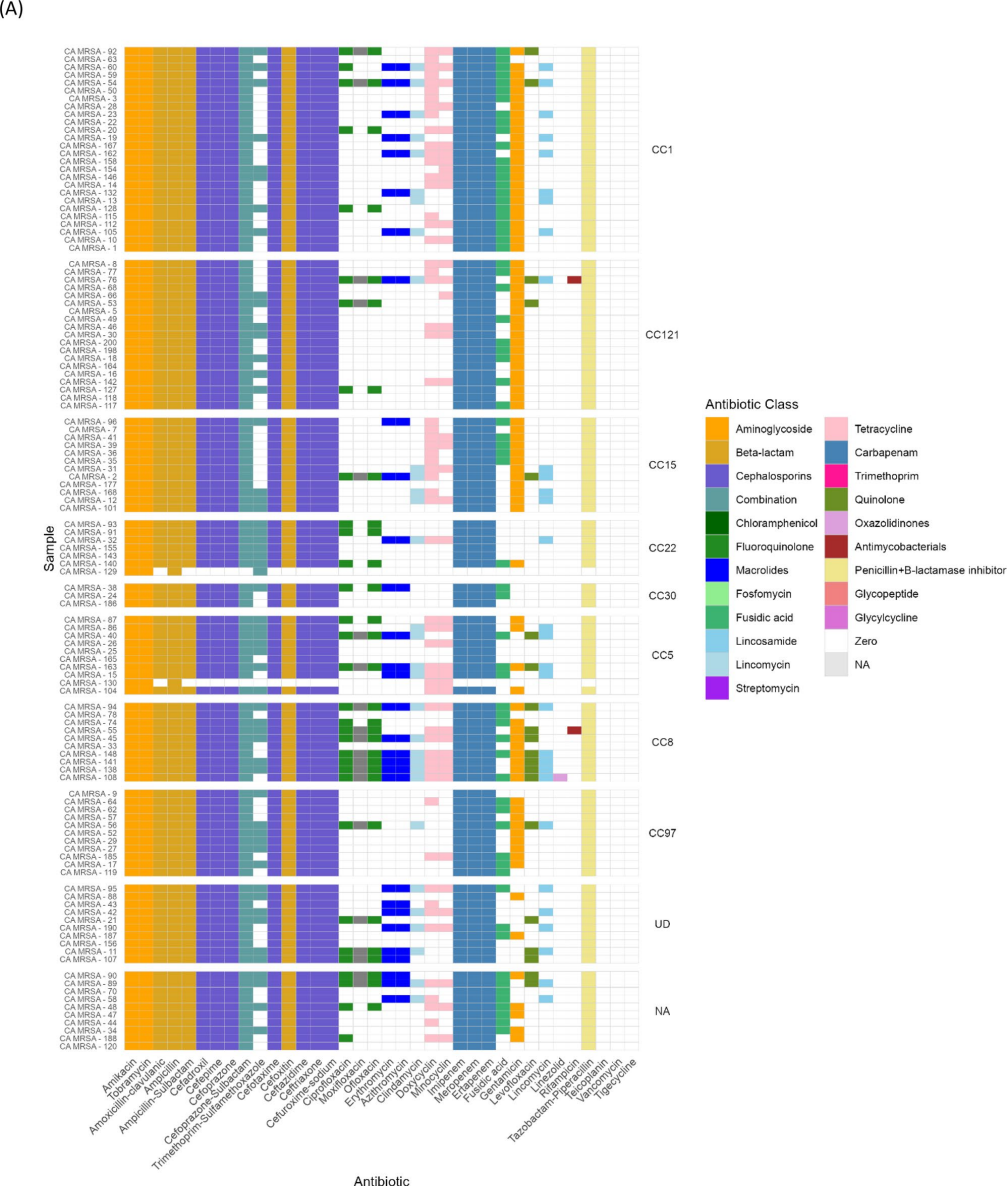

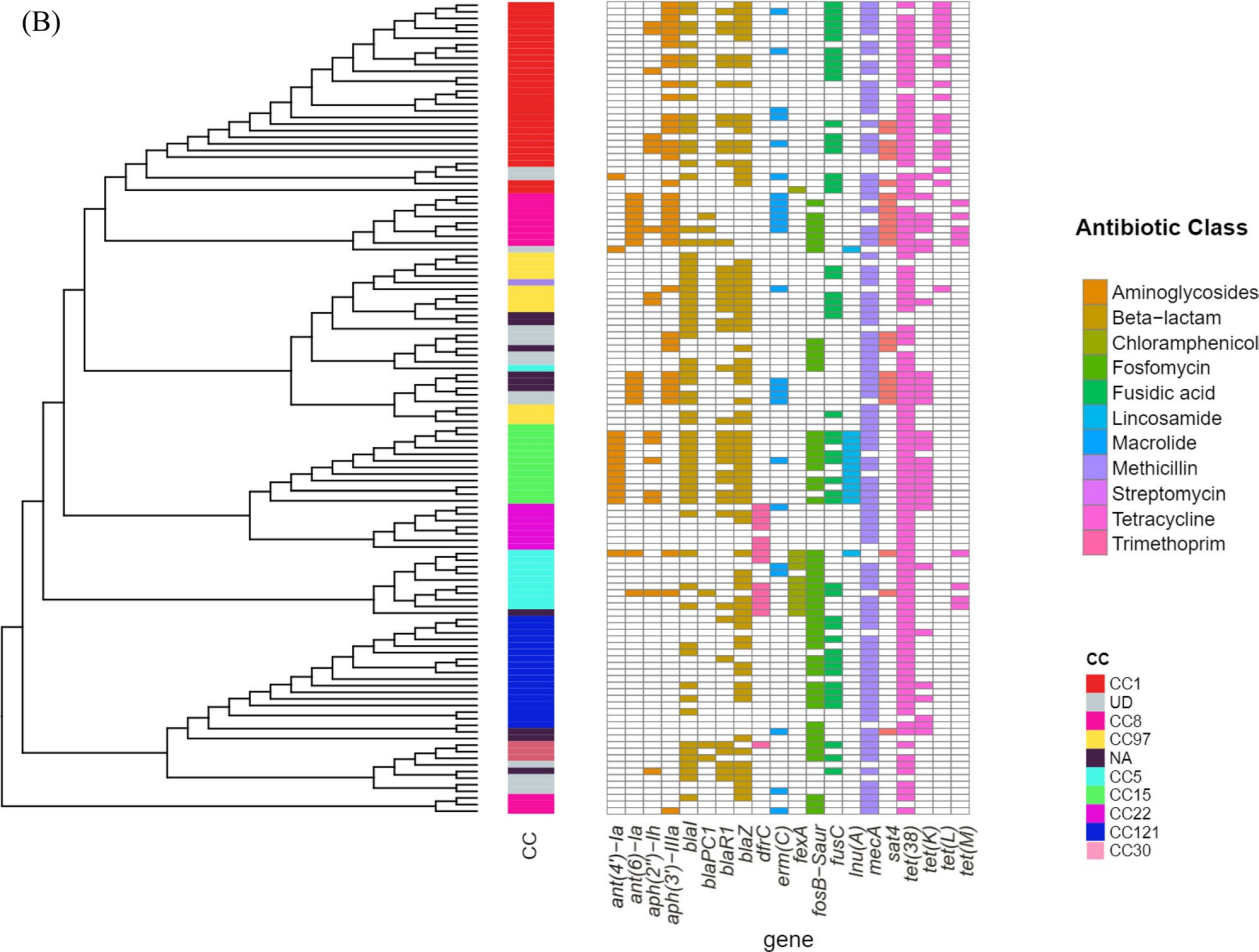

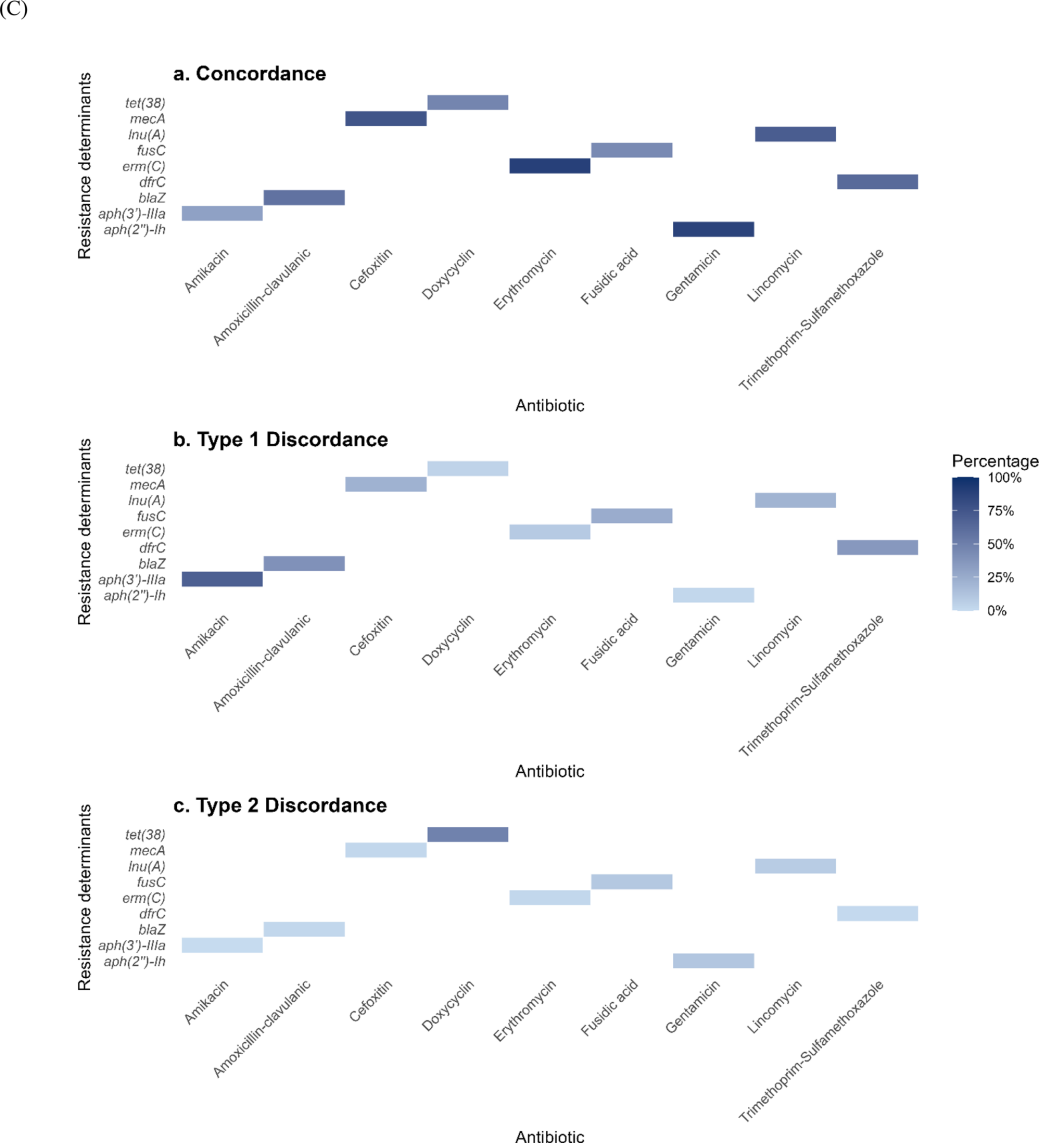
(**A**) Presence and Absence Matrix of AMR phenotypes across CA- MRSA Clonal complexes (CCs) in Egypt. The heatmap displays the phenotypic resistance profiles to various antibiotics (listed on the x-axis) across different CA-MRSA clonal complexes (listed on the y-axis). Antibiotics are grouped by class and color-coded in the legend. (**B**) Hierarchical clustering dendrogram illustrating antimicrobial resistance (AMR) profiles and their association with resistance determinants across screened isolates. Isolates are grouped based on similarity in AMR gene presence/absence patterns. Colored vertical bars adjacent to the dendrogram indicate clonal complexes (CCs), with each color representing a distinct CC. Resistance genes are color-coded by antibiotic class for clear differentiation. (**C**) Heatmap displaying the concordance and discordance between AMR genes and phenotypic susceptibility profiles across various antibiotics. Each row corresponds to an AMR gene, and each column to an antibiotic. (a) Concordance indicates cases where the presence of an AMR gene aligns with observed resistance (b) Type 1 discordance indicates cases with phenotypic resistance but no detected resistance gene (c) Type 2 discordance indicates cases with a resistance gene but no corresponding phenotypic resistance. Color intensity represents the percentage of agreement or disagreement, with shades ranging from dark blue (high) to light blue (Low)

We evaluated AMR phenotypes among the screened CA-MRSA isolates, all of which were classified as MDR, showing non-susceptibility to at least three different antibiotic classes (Fig. 2A, Supplementary file 4). Notably, all isolates remained susceptible to teicoplanin, vancomycin, and tigecycline. All isolates consistently exhibited resistance to penicillins, aminoglycosides, cephalosporins, and carbapenems.

Methicillin resistance was initially determined phenotypically based on resistance to cefoxitin according to CLSI guidelines (CLSI M100). A total of 121/123 isolates demonstrated phenotypic resistance to cefoxitin, while two isolates were categorized as susceptible. Genotypic characterization of methicillin resistance was subsequently performed by screening for the *mecA* gene which was detected in 96 isolates. The resistance patterns revealed complete resistance to amikacin and tobramycin (100%), as well as high-level resistance to cefoxitin (98%) and ampicillin-sulbactam (98%). Resistance was also high against carbapenams, including imipenem (98%), meropenem (98%), and ertapenem (98%). In contrast, the isolates showed higher susceptibility to fluorquinolones, with ciprofloxacin (73%), moxifloxacin (83%), and ofloxacin (75%), demonstrating moderate resistance rates. Additionally, macrolides such as erythromycin and azirthomycin exhibited 75% resistance, and lincomycins, such as clindamycin showed a resistance rate of 73%. Among the tetracyclines, doxycycline and minocycline showed moderate susceptibility (49% and 62%, respectively), but all isolates remained fully susceptible to glycopeptides (teicoplanin and vancomycin) and tigecycline. Notably, CC8 exhibited significant resistance to multiple antibiotic classes, with strong associations observed for fluoroquinolone resistance (Ofloxacin: *P* = 6.93E-05, OR = 23.66; Ciprofloxacin: *P* = 0.00016, OR = 19.64) and macrolide resistance (Erythromycin and Azithromycin: both *P* = 0.006, OR = 7.19). This MDR profile clearly distinguishes CC8 from other clonal complexes. Resistance to fusidic acid showed a significant positive association with CC1 (*P* = 0.000171, OR = 11.48) and a significant negative association with CC22 (*P* = 0.003, OR = 0), indicating potential lineage-specific trends. Additionally, linezolid resistance showed a weak positive association with CC8 (*P* = 0.101, OR = ∞), suggesting potentially clonal distribution of this resistance phenotype (Supplementary file 10).

We then evaluated AMR genotypes among CA-MRSA isolates. All isolates were MDR, with 37 distinct resistance genes identified (Fig. 2B, Supplementary file 5). Key resistance genes included *mecA* (encoding for methicillin resistance) 78% of isolates, *tet(38)* (encoding for tetracycline resistance) in 89.4% of isolates, and *ermC* (encoding for erythromycin resistance) in 22%. The β-lactamase operon (*blaZ*, *blaI*, *blaR*) was common, with *blaZ* found in 60.2% of isolates. The *dfrC* gene (encoding for trimethoprim resistance) was present in 12.2% of isolates, mostly in CC5 and CC22. The *fexA* gene (encoding for chloramphenicol resistance) was found in CC5 and CC1. Notably, the *fusC* gene (encoding for fusidic acid resistance) was detected in 38.7% of isolates, including CC30, CC121, CC5, CC15, CC97, and CC1, with eight isolates carrying SCC*mec* VI.

The *fosB* gene, which encodes for fosfomycin resistance, was present in 42.3% of isolates, especially in CC121 (12%), CC5 (7%), CC8 (7%), CC15 (7%), and CC30 (2%). Tetracycline resistance was mainly driven by *tet(38)*, while *tetM* and *tetK* were found in 7% and 25% of isolates, respectively. Interestingly, *tetM* was absent in CA-MRSA clones such as CC1 and CC121, whereas *tetK* was found primarily in CC8 and CC5. CC8 was split between ST8 and ST239, with the latter predominating (54.5%). ST239 isolates were associated with *spa* type t037 and SCC*mec* V/III, while ST8 isolates carried SCC*mec* V/III with a predominance of SCC*mec* V. CC97 isolates showed minimal resistance, restricted to *blaZ*, *mecA*, and *tet(38)*, but 45% exhibited fusidic acid resistance (*fusC*). These CC97-MRSA isolates were further divided into *fusC+* subtypes (CC97-MRSA-V and CC97-MRSA-I).

A strong association between clonal lineages and MDR profiles was observed (Fig. 2B). Statistical analysis of AMR gene distribution across CCs revealed distinct resistance profiles and significant associations. CC1 was strongly associated with the presence of *tet(L)* (*P* = 2.60E-12, OR = 131.6) and showed complete absence of *fosB* (*P* = 1.18E-09). CC15 exhibited highly significant associations with both *ant(4’)-Ia* and *lnu(A)* (*P* = 1.01E-12, OR = 583.8 for both). The *fexA* gene was significantly associated with CC5 (*P* = 2.13E-09, OR = 262.6), while CC8 was strongly linked to *ant(6)-Ia* (*P* = 3.76E-08, OR = 97.6) and negatively associated with *fusC* (*P* = 0.000916, OR = 0). CC22 showed significant enrichment for *dfrC* (*P* = 9.43E-06, OR = 66.5), and CC30 displayed the highest odds ratio for *bla*_*PC1*_ (*P* = 0.008, OR = 40.5) (Supplementary file 9).

Next, we analyzed the association between resistance determinants and resistance phenotypes, including acquired genes, gene mutations, and phenotypic resistance, across seven antibiotic classes (Fig. 2C). Concordance analysis revealed notable trends in the concordance and discordance between genotypic and phenotypic resistance profiles. Concordance was high for specific resistance genes, such as *mecA* (76%) for methicillin resistance and *ermC* (89%) for erythromycin resistance, indicating that the presence of these genes was predictive of resistance to these antibiotics. However, significant discordance (type 1) was observed for some aminoglycosides, such as amikacin with *aph(3’’)-IIIa* gene (51%), indicating phenotypic resistance in the absence of a corresponding resistance gene.

### CA-MRSA predicted to exhibit high virulence

**Fig. 3:**
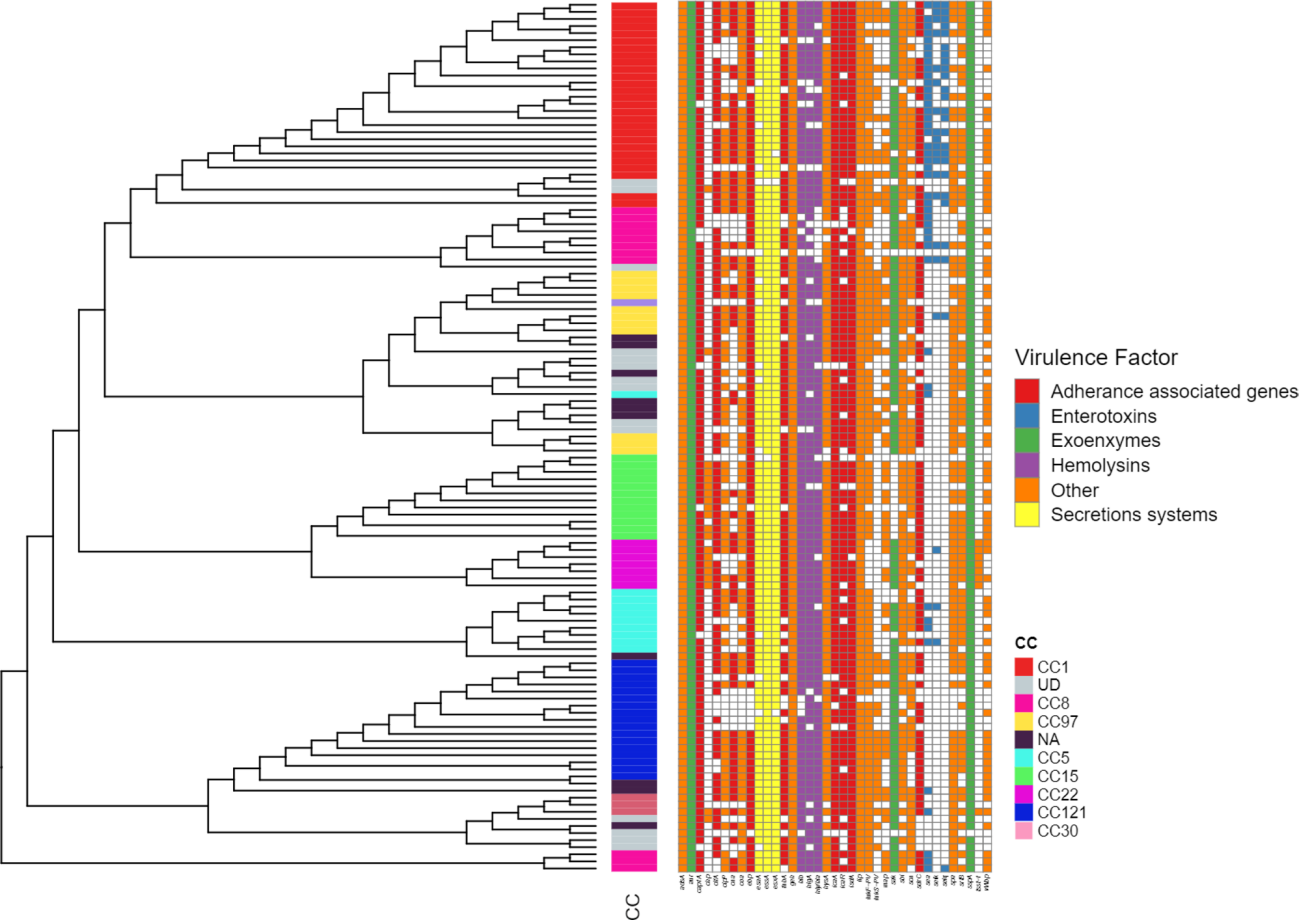
Hierarchical clustering dendrogram illustrating virulence gene profiles and their association with virulence determinants across screened isolates. Isolates are grouped based on similarity in virulence gene presence/absence patterns. Colored vertical bars adjacent to the dendrogram indicate clonal complexes (CCs), with each color representing distinct CC. Virulence genes are color-coded by antibiotic class for clear differentiation

**Fig. 4:**
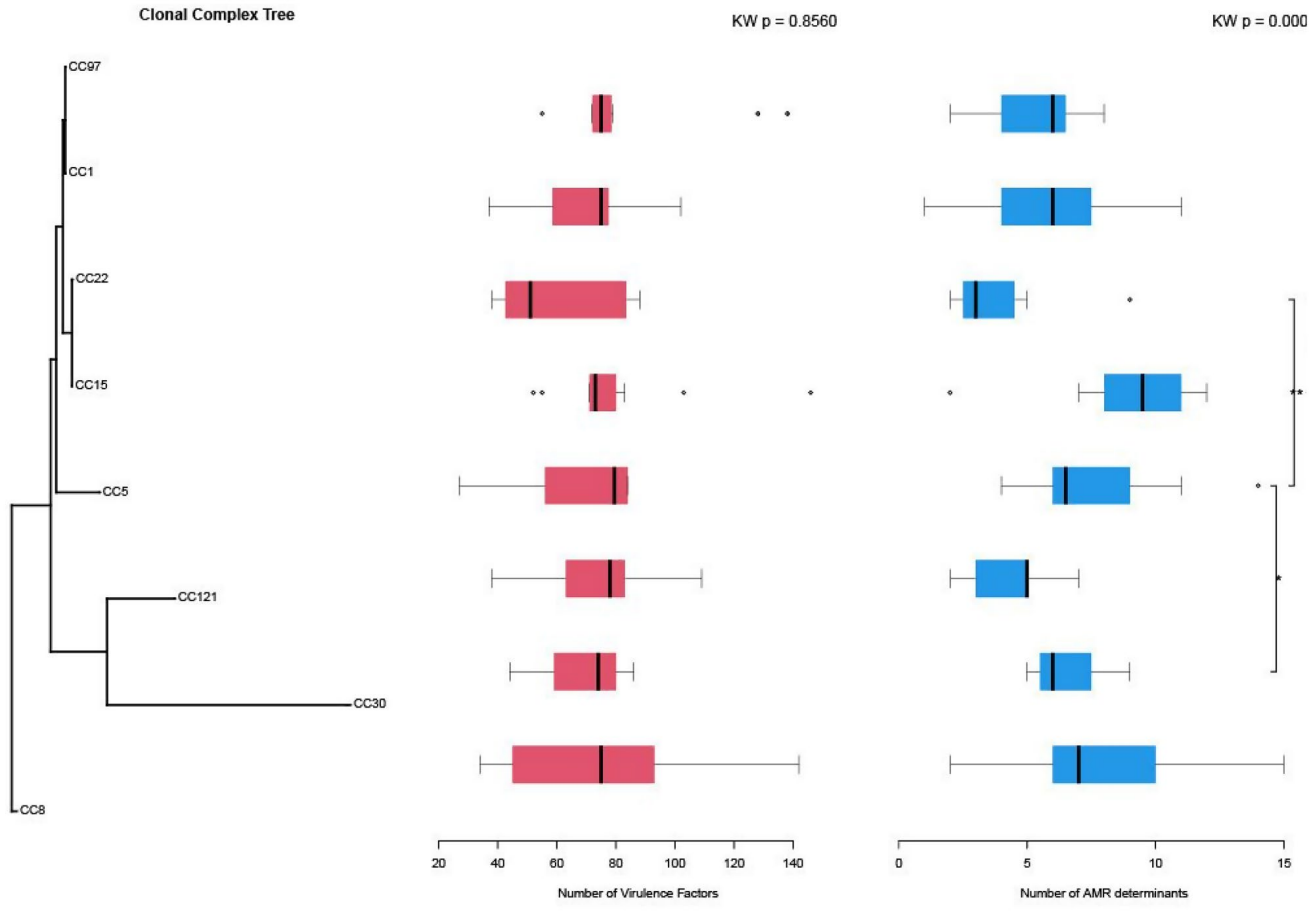
Boxplots illustrating the distribution of virulence factors and AMR determinants across clonal complexes (CCs) within CA-MRSA isolates. The phylogenetic tree on the left depicts the evolutionary relationships among the CCs. Core genome alignments were generated using Parsnp, filtered with Gblocks, and used to construct a maximum-likelihood tree in RAxML under the GTR+GAMMA model with 1,000 bootstrap replicates. Branch lengths reflect evolutionary distances (substitutions per site). The middle panel presents the number of virulence factors (shown in red) associated with each CC, while the right panel displays the corresponding number of AMR determinants (in blue). Each boxplot summarizes the distribution within a CC: the horizontal line indicates the median, boxes represent the interquartile range (IQR), and whiskers extend to the most extreme data points within 1.5 times the IQR from the quartiles. Data points outside this range are shown individually as outliers. All individual values are plotted, with whiskers denoting the minimum and maximum within the IQR threshold

Analysis of the virulome of CA-MRSA isolates revealed a wide range of virulence-related genes, with the total number of virulence factors per isolate varying from 27 to 225 (Supplementary file 6). These genes were classified into five major categories: adherence-associated genes, exoenzymes, immune evasion factors, secretion systems, and toxins (Fig. 3). However, the number of predicted virulence factors did not differ significantly among clonal complexes (KW *p* = 0.8560), indicating a relatively uniform distribution of virulence-associated genes across lineages. CC8, CC5, and CC30 exhibited the highest median numbers of virulence genes (~ 85 and ~ 80, respectively). CC22 also displayed a relatively high abundance of virulence genes (~ 78). In contrast, CC1, CC121 (community-associated lineages), and CC15 exhibited lower median virulence gene counts, approximately 65, 68, and 67, respectively. In contrast, the number of AMR determinants varied significantly between clonal complexes (KW *p* = 0.00020). CC8 showed the highest enrichment with a median of approximately 10 genes, followed by CC1 and CC5 with medians of around 7 and 6, respectively. In contrast, CC15 had a lower AMR gene burden, with a median of approximately 4, and was not among the most enriched clonal complexes. CC8, emerged as the most concerning, showing both high virulence (~ 85 factors) and a moderate number of AMR determinants (~ 12). CC97, a livestock-associated clone, harbored a moderate number of AMR determinants (~ 8) and a moderate number of virulence genes, with ~ 65 virulence genes identified (Fig. 4).

Key virulence factors included Panton-Valentine leukocidin (PVL), which was present in most clones, though isolates from CC22, CC1, CC121, CC15 and CC8(13.82%,*n* = 17) lacked PVL genes. Enterotoxins (e.g., *sea*, *seb*) were detected in 36%–20% of isolates; mainly CC1, CC8, CC5 and CC121 while the toxic shock syndrome toxin (*tsst*) was absent in most strains, except for eight isolates from CC22 and CC30. Exoenzymes, such as aureolysin (*aur*) and serine proteases (*sspA*, *sspB*), were highly prevalent in all clones, as were secretion system genes (*esaA*, *esxA*) and capsular serotype 8 (*cap8*) genes. Immune evasion factors, including *adsA* and *sbi*, as well as adhesion genes (*ebp*, *sdrC*, *ica*), were also widespread across clones. Notably, iron uptake proteins (*isdA*, *isdB*) were found in most clones (CC1, CC121, CC15, CC22, CC30, CC8 and CC97), while adhesion factors such as *clfA* and *fnbA* were prevalent, though some variability was observed across CCs.

The immune evasion cluster (IEC) system was prevalent across most clonal complexes. IEC type E (*scn*, *sak*) was the dominant, detected in 62% of isolates and distributed across CC97, CC5, CC8, CC1, CC121, CC22 and CC30. IEC type D (*scn*, *sak*,* sea*) was less common, occurring in 26% of the isolates and distributed across CC5, CC1, CC121 and CC8. Notably, eight isolates, predominantly belonging to CC5, CC8, and CC15, were negative for IEC genes (Fig. 4; Supplementary File 6).

### Virulence genes are mobile in the accessory genome

**Fig. 5:**
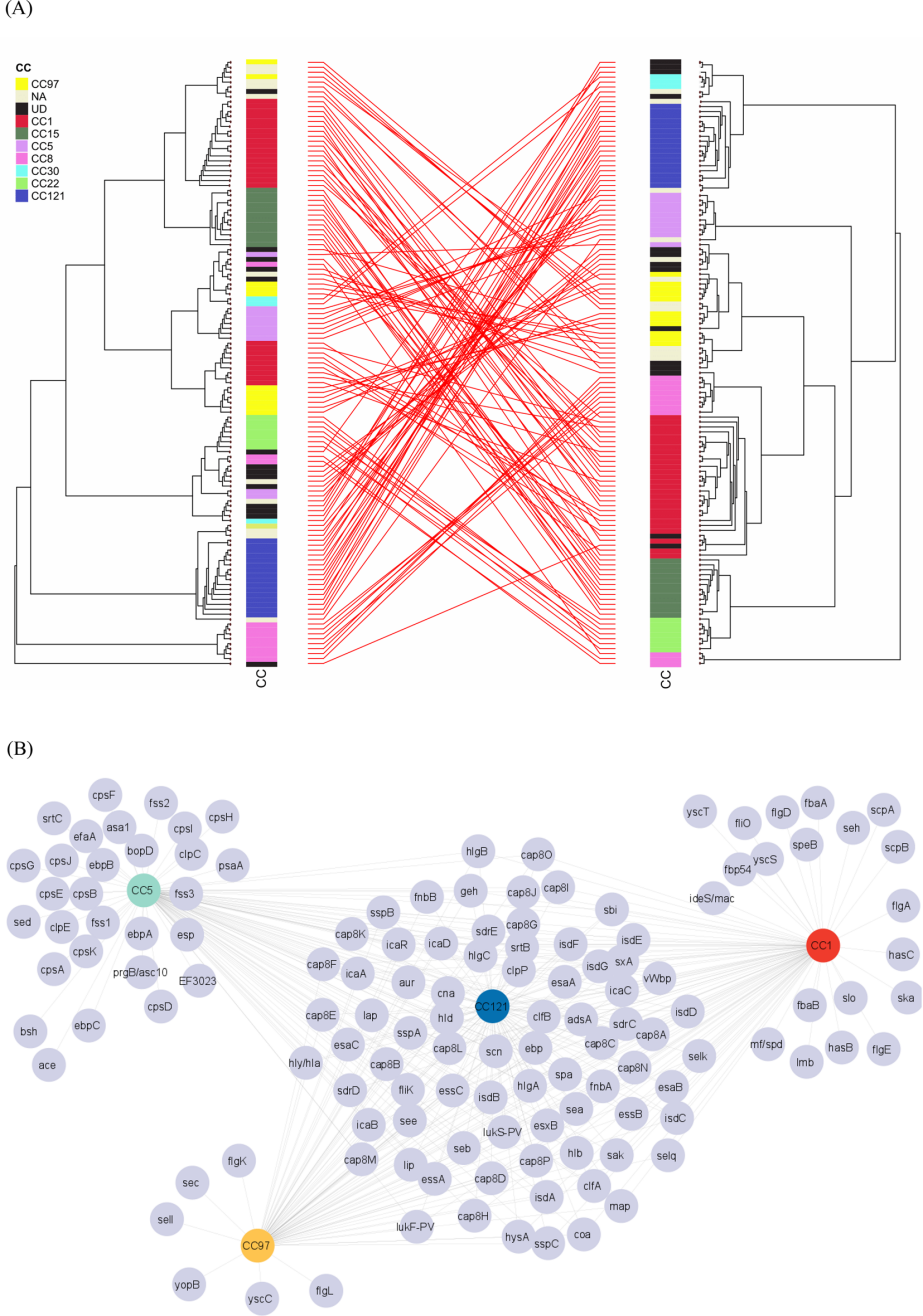
(**A**) Tanglegram showing comparative co-phylogeny of CA-MRSA isolates analyzed using core genome SNP and pangenome regarding the clonal complexes. The left panel shows the phylogenetic tree based on core genome single-nucleotide polymorphisms (SNPs), reflecting the genetic relationships among the isolates. The right panel displays the phylogenetic tree based on the pangenome, representing the distribution of accessory genes across the isolates. Lines between the two phylogenies connect clonal complexes, illustrating shifts between core and pangenome-based classifications. (**B**) Network representation of shared virulence genes among selected CCs. Colored nodes denote clonal complexes: CC1 (red), CC5 (green), CC97 (yellow), and CC121 (blue), while gray nodes represent individual virulence genes. Edges indicate the presence of a given gene in at least one isolate belonging to the connected CC. This bipartite network visualizes both shared and unique virulence gene profiles, highlighting patterns of gene co-occurrence across lineages without implying direct gene flow or horizontal transfer. CC1, CC5, and CC121 were included based on their higher representation in the dataset and the notable diversity in their virulence gene content. The network is intended to illustrate general trends in gene distribution across clonal lineages, rather than to infer evolutionary relationships or mechanisms

To explore the evolutionary dynamics of the screened isolates, we compared phylogenetic relationships based on core genome SNPs versus the pangenome (Fig. 5A). The core genome analysis clustered isolates into six major clades: CC1 and CC8, CC97, CC15 and CC22, CC5, CC121 and CC30, and a distinct CC8 group. In contrast, pangenome classification revealed more complex diversity, with seven clades and a higher variability across clonal complexes. CC121, for instance, appeared more distantly related to CC30 in the pangenome analysis, although they were closely related in the core genome.

Additional variability is observed within clonal complexes CC97, CC1, and CC5 (Fig. 5A). Unlike our phylogeny based on core genome SNPs, CC97 isolates did not cluster together and were distributed across three groups. In contrast, CC8 isolates exhibited greater intra-lineage diversity in the core genome than in the pangenome, suggesting that members of this globally disseminated clonal complex may share key accessory genes despite underlying core genomic variation.

Among the screened collection, the core genome comprised 1,347 genes (27.9%), while the accessory genome accounted for 3,475 genes (72.1%). Of the accessory genes, 2,917 were present in at least 60.4% of the isolates (Supplementary file 2). A network analysis of virulence gene presence among key clonal complexes identified CC121 as a central node, sharing virulence genes with CC1, CC97, and CC5 (Fig. 5B). Fewer, specific virulence genes were shared among CC1 and CC121 isolates. In contrast, CC97 isolates were more frequent and shared virulence genes with multiple other CCs. CC5 had several connections, indicating a closely related gene network. Notably, CC121 harbored the highest number of unique virulence genes, including those for biofilm formation (*icaA*,* icaC*) [17], immune evasion (*scn*,* spa*) [42], and surface proteins (*clfA*,* clfB*,* cap*) [50]. CC97 had fewer unique virulence factors, primarily related to secretion systems (*sec*,* yopB*, and *yscC*) [51] and flagellar assembly (*flgK*,* flgL*) [52]. CC1 contained genes for cytotoxicity (e.g., *slo*, *ska*) [53] and immune evasion (*scpA*, *scpB*, *speB*) [54], while CC5 carried genes associated with capsule synthesis (*cpsA-E*) and adhesion (*ebpA-C*, *efaA*). A similar analysis, grouped by different hosts, identified CC5 and CC97 as the primary toxigenic complexes. CC97 carried the classical enterotoxin genes *sec* and *see*, while CC5 possessed *seh*, a non-classical enterotoxin gene [55]. CC121 also harbored the non-classical enterotoxin, *see* gene [55].

## Discussion

This study provides one of the first comprehensive genomic analyses of CA-MRSA in Egypt, revealing extensive genetic and phenotypic diversity within a localized population. Whole-genome sequencing of 123 isolates collected from Alexandria, Egypt identified eight major CCs, encompassing both globally disseminated lineages (CC1, CC5, CC8, CC22, and CC121) and regionally specific variants, including five novel sequence types (ST8157–ST8161). These findings highlight the coexistence of international MRSA clones with locally evolved sub-lineages and suggest that Alexandria acts as a convergence point for MRSA diversity in North Africa [56, 57].

The coexistence of healthcare-associated (HA-MRSA) and community-associated (CA-MRSA) clones, particularly CC5, CC8 (ST239/ST8), and CC22 (ST22-IV), illustrates the erosion of traditional epidemiological boundaries between hospital and community environments. This overlap likely reflects patient movement, unregulated antibiotic use, and environmental exposure, creating opportunities for horizontal gene transfer and selection of MDR strains [58]. Notably, livestock-associated CC97 was also detected among human isolates, supporting previous evidence of zoonotic exchange and adaptation across ecological niches [6, 59, 60]. Together, these results demonstrate the dynamic and interconnected population structure of MRSA in Egypt, shaped by antibiotic pressure, mobility, and genetic recombination.

Antimicrobial susceptibility testing and genomic screening revealed widespread multidrug resistance across all isolates. Methicillin resistance was initially determined phenotypically based on cefoxitin resistance and subsequently confirmed genotypically by screening for the *mecA* gene. The high prevalence of *mecA* and *blaZ* confirms the dominance of β-lactam resistance mechanisms, while the frequent detection of *fosB* (42%) and *fusC* (39%) is particularly concerning. *FosB* encodes an enzyme that inactivates fosfomycin, a drug increasingly used as a last-line treatment for MRSA infections, while *fusC* confers resistance to fusidic acid, a topical antibiotic widely available without prescription in Egypt [58, 61]. The co-occurrence of these genes in multiple clonal complexes suggests a selective advantage under community antibiotic pressure.

The predominance of *SCCmec* types IV and V among CA-MRSA lineages (especially CC1 and CC121) contrasts with the presence of *SCCmec* types III and VI within CC8 and CC5, typically associated with HA-MRSA [62]. This pattern indicates ongoing gene flow between healthcare and community populations. Furthermore, the identification of *fusC* within multiple *SCCmec* backgrounds implies mobilization across distinct lineages, reinforcing its epidemiological importance in the region [63, 64].

Concordance analysis between phenotypic and genotypic resistance profiles revealed high agreement for *mecA* (76%) and *ermC* (89%), confirming the predictive value of genomic screening for β-lactam and macrolide resistance. However, notable genotype–phenotype discordance was observed for certain aminoglycosides (e.g., amikacin), likely due to undetected resistance mechanisms, gene regulation differences, or borderline oxacillin-resistant *S. aureus* (BORSA) phenotypes [65]. These findings underline the continued importance of combining molecular and phenotypic testing to capture the full spectrum of resistance mechanisms in clinical isolates.

Analysis of the virulome revealed extensive diversity in virulence gene content across clonal complexes, although overall virulence gene counts did not differ significantly among lineages (KW *p* = 0.8560). This suggests that while the genetic potential for virulence is widespread, its expression may be modulated by regulatory variation rather than simple gene presence. CC8, CC5, and CC30 exhibited the highest median counts of virulence genes, consistent with their association with invasive infections. In contrast, community-associated lineages CC1, CC15, and CC121 harbored fewer virulence determinants but may compensate through enhanced transmission efficiency and environmental persistence [66].

The network analysis of virulence factors revealed both shared and lineage-specific gene profiles. CC121 emerged as a central node connecting multiple lineages, carrying genes linked to biofilm formation (*icaA*, *icaC*), immune evasion (*scn*, *spa*), and adhesion (*clfA*, *clfB*, *cap*) [17, 50, 52]. CC97 isolates encoded genes such as *yopB* and *yscC*, typically associated with *Yersinia* species, suggesting horizontal gene transfer that may expand host range. CC5 carried capsule synthesis (*cpsA–E*) and adhesion (*ebpABC*) genes, consistent with its persistence in healthcare settings. These data highlight the mosaic nature of the *S. aureus* accessory genome and its role in shaping adaptation to specific ecological and host contexts.

Comparative phylogenetic analysis of core and accessory genomes further supports the modular and mobile nature of virulence and resistance determinants. The tanglegram comparison revealed substantial incongruence between the core-genome and pangenome trees, particularly within CC97, CC1, and CC5, indicating frequent recombination or horizontal gene transfer. This decoupling between phylogenetic relatedness and accessory gene content emphasizes that MRSA evolution is not purely clonal but shaped by extensive gene exchange across lineages [67].

The genomic diversity observed among isolates from Alexandria mirrors patterns observed globally, with community, hospital, and livestock-associated MRSA clones co-circulating within the same urban setting. The predominance of CA-MRSA lineages such as CC1-SCCmecV and CC121-SCCmecV, coupled with the detection of pandemic HA-MRSA clones (ST239, ST22), demonstrates that global dissemination and local adaptation are occurring concurrently [56, 58, 68]. Alexandria’s unique geographical and socio-economic profile likely contributes to this genomic admixture. As Egypt’s principal Mediterranean port and a rapidly expanding industrial and commercial hub, the city experiences intense human mobility, maritime trade, and industrial development [69]. Environmental contamination from untreated wastewater and industrial effluents may foster the persistence and exchange of antibiotic-resistant bacteria in aquatic and urban environments. Overcrowding, combined with widespread over-the-counter antibiotic access, further facilitates community-level selection and transmission of resistant clones [70].

Together, these factors create a highly connected ecological interface linking hospitals, households, and environmental reservoirs, conditions under which both global MRSA lineages and locally evolving variants can persist and spread. The elevated resistance to fusidic acid and fosfomycin observed in this study reflects these pressures, underscoring the urgent need for strengthened antimicrobial stewardship and environmental regulation in urban Egypt.

Although this study provides a valuable genomic baseline for MRSA circulating in the region, it has several limitations. The dataset represents a single urban area and a defined time period and therefore may not capture temporal or spatial variation across Egypt. We acknowledge that the collection of isolates during the peak of the COVID-19 pandemic could raise concerns about potential bias in observed MRSA prevalence and resistance patterns. However, several studies comparing MRSA rates before, during, and after the pandemic suggest that, while local and temporal variations exist, there has not been a consistent global increase in MRSA prevalence attributable solely to pandemic conditions. While random sampling was used to reduce bias, the study design does not allow inference of transmission chains or population dynamics. Assembly fragmentation in a subset of isolates may also have limited fine-scale detection of mobile genetic elements. Future studies should incorporate broader sampling, temporal data, and integration with clinical metadata to better understand transmission pathways, infection outcomes, and regional trends in resistance evolution.

In summary, CA-MRSA isolates from Alexandria, Egypt exhibit a complex population structure comprising both globally disseminated and locally evolved lineages. The widespread presence of *fusC*, *fosB*, and other multidrug-resistance determinants, combined with diverse virulence repertoires and evidence of gene exchange between community, hospital, and livestock reservoirs, underscores the adaptive potential of MRSA in Egypt. Alexandria’s dense population, industrial expansion, and environmental connectivity provide fertile conditions for the persistence and spread of resistant clones. These findings position the Egyptian MRSA population within the global evolutionary landscape and underscore the need for integrated genomic surveillance within a One-Health framework to inform infection control and antimicrobial stewardship.

## Supplementary Information

Below is the link to the electronic supplementary material.

Supplementary Material 1: Supplementary file 1: Assembly statistics for 123 CA-MRSA isolates. Supplementary file 2: Pie chart for Pangenome analysis. Supplementary file 3: Typing results for 123 CA-MRSA isolates. Supplementary file 4: Association between phenotypic antimicrobial resistance patterns and clonal complexes among CA-MRSA isolates. Supplementary file 5: Distribution of antibiotic resistance profiles among the 123 CA-MRSA isolates. Supplementary file 6: Distribution of virulence profiles among the 123 CA-MRSA isolates. Supplementary file 7: Individual accession numbers of CA-MRSA isolates. Supplementary file 8: Distribution of antibiotic resistance genes across CA MRSA sequence types (STs) in Egypt. Supplementary file 9: Association between CA-MRSA clonal complexes (CCs) and antimicrobial resistance genotypes. Supplementary file 10: Association between CA-MRSA isolates clonal complexes (CCs) and antimicrobial resistance phenotypes

## Data Availability

Short-read data are available from the National Center for Biotechnology Information (NCBI) Sequence Read Archive, associated with BioProject PRJNA1135495 (https://dataview.ncbi.nlm.nih.gov/object/PRJNA1135495?reviewer=bu3i74l6hj8ocq34g3o4cf8bk7). Assembled genome data and individual accession numbers are provided in Supplementary File 7.

## Abbreviations

Agr: Accessory gene regulator
AMR: Antimicrobial resistance
CA-MRSA: Community associated MRSA
CCs: Clonal complexes
CDC: Center of disease control and prevention
dfrC: Dihydrofolate reductase type 1
QS: Quorum sensing
HA-MRSA: Hospital associated MRSA
LA-MRSA: Livestock associated MRSA
lnu (A): Lincosamide-nucleotidyltransferase
MGEs: Mobile genetic elements
Mini-BAL: Mini bronchoalveolar lavage
MLST: Multi-locus sequence typing
MRSA: Methicillin-resistant *S. aureus*
PVL: Panton-Valentine leucocidin
*S. aureu*s: *Staphylococcus aureus*
STs: Sequence types
TET: Tetracycline
WGS: Whole genome sequencing
WHO: World health organization
